# The Immunosuppressive Properties of the HIV Vpr Protein Are Linked to a Single Highly Conserved Residue, R90

**DOI:** 10.1371/journal.pone.0005853

**Published:** 2009-06-10

**Authors:** Irina Tcherepanova, Aijing Starr, Brad Lackford, Melissa D. Adams, Jean-Pierre Routy, Mohamed Rachid Boulassel, David Calderhead, Don Healey, Charles Nicolette

**Affiliations:** 1 Research and Development Department, Argos Therapeutics, Inc. Durham, North Carolina, United States of America; 2 Duke Clinical Research Institute, Durham, North Carolina, United States of America; 3 National Institute of Environmental Health Sciences, National Institutes of Health, Research Triangle Park, North Carolina, United States of America; 4 BD Diagnostics, Durham, North Carolina, United States of America; 5 McGill University Health Center and INSERM Unit 743, Montréal, Canada; Comprehensive AIDS Reseach Center, China

## Abstract

**Background:**

A hallmark of AIDS progression is a switch of cytokines from Th1 to Th2 in the plasma of patients. IL-12, a critical Th1 cytokine secreted by antigen presenting cells (APCs) is suppressed by Vpr, implicating it as an important virulence factor. We hypothesize that Vpr protein packaged in the virion may be required for disabling APCs of the first infected mucosal tissues. Consistent with this idea are reports that defects in the C-terminus of Vpr are associated with long-term non-progression.

**Principal Findings:**

Vpr RNA amplified from various sources was electroporated into monocyte-derived DC and IL-12 levels in supernatants were analyzed. The analysis of previously reported C-terminal Vpr mutations demonstrate that they do not alleviate the block of IL-12 secretion. However, a novel single conservative amino acid substitution, R90K, reverses the IL-12 suppression. Analysis of 1226 Vpr protein sequences demonstrated arginine (R) present at position 90 in 98.8%, with other substitutions at low frequency. Furthermore, none of sequences report lysine (K) in position 90. Vpr clones harboring the reported substitutions in position 90 were studied for their ability to suppress IL-12. Our data demonstrates that none of tested substitutions other than K relieve IL-12 suppression. This suggests a natural selection for sequences which suppress IL-12 secretion by DC and against mutations which relieve such suppression. Further analyses demonstrated that the R90K, as well as deletion of the C-terminus, directs the Vpr protein for rapid degradation.

**Conclusion:**

This study supports Vpr as an HIV virulence factor during HIV infection and for the first time provides a link between evolutionary conservation of Vpr and its ability to suppress IL-12 secretion by DC. DC activated in the presence of Vpr would be defective in the production of IL-12, thus contributing to the prevailing Th2 cytokine profile associated with progressive HIV disease. These findings should be considered in the design of future immunotherapies that incorporate Vpr as an antigen.

## Introduction

Human immunodeficiency virus I (HIV- I) possesses many weapons to evade the immune system of an infected individual. High sequence variability of the HIV genome allows viral escape from both humoral and cellular immune responses. HIV mutations leading to CTL escape are attributed to the failure of cellular immunity to control HIV infection [1]. Recent studies demonstrating an inverse correlation between the frequency of CTL escape via mutation of HIV antigens and the replicative capacity of the virus further confirm this idea [2]. Mutations that evade inhibition of compounds comprising HAART regimens are also well documented.

In addition to direct evasion of immune responses through mutagenesis, HIV also disturbs cytokine profiles in the plasma of HIV patients, thereby impeding effective immune responses against the infection, a feature which is receiving more recognition [3]. The cytokine response to an invading microorganism is critical for priming DC-mediated adaptive immune responses and is subject to tight regulation, particularly in the case of Th-1 polarizing cytokines [4], [5]. During early HIV infection Th 1 cytokines are detected in the plasma of infected individuals, however, at later stages of disease, the cytokine profile switches to a Th 2 profile indicative of a decay in the antiviral immune response [6]. One of the cytokines associated with Th 1 polarization is IL-12 and it has been reported that its level is decreased in HIV-positive patients versus healthy individuals [7]. The association of IL-12 with productive CD8-mediated cytolytic activity is well documented in tumor models *in vitro* and in human clinical trials [8], [9]. Likewise, the impaired immune response to HIV was shown to be restored *in vitro* by addition of exogenous IL-12 underscoring the critical importance of this cytokine [10].

IL-12 is produced by activated antigen presenting cells, macrophages, and dendritic cells and its level can be modulated by infection of those cells types with HIV. The viral protein R (Vpr) is thought to contribute to this effect. Monocyte and DC cultures incubated in the presence of extracellular Vpr were shown to downregulate CD80, CD83, and CD86 in these cell types, blocking their activation and maturation [11]. Another study supports the observation that Vpr impairs expression of CD80, CD83, and CD86 costimulatory molecules as well and documents that Vpr inhibits IL-12 production and upregulates IL-10 cytokine secretion by DC [12]. That study implicated Vpr as an important virulence factor in HIV infection and suggested that the suppressed immune responses may be a consequence of Vpr-mediated block of IL-12 production by DC. The association of Vpr mutations with long-term non-progressor (LTNP) status is also consistent with the idea of Vpr as a virulence factor [13]–[15].

Approximately 275 molecules of Vpr protein are incorporated into HIV virions released form productively infected cells [16]. Therefore, Vpr is present during early stages of infection and available to rapidly exert its function on the cells it first encounters, DC of the mucosal membranes. While integrity of the α-helices of Vpr (residues 35–46) is required for efficient packaging into virions [17]. it is the C-terminus that is implicated in its virulent activity after release into the infected cell. The mitochondrial domain located within the C-terminus, specifically amino acid 77, is implicated in mitochondrion-dependent apoptosis [13], [18]. Other reports indicate that the LXXLL domain located in the C-terminus is responsible for the suppressive effect on IL-12 via a glucocorticoid-like mechanism [19].

The present study explored the effect of Vpr expressed from translation-competent RNA electroporated into DC. Here we confirm earlier the observations that Vpr suppresses IL-12 secretion by DC and further explore the involvement of the Vpr C-terminus in IL-12 suppression as an important HIV virulence factor. Detailed analysis using previously described mutations within the C-terminus identified a single point mutation, R90K, which can prevent the IL-12 blockade.

## Results

### Vpr suppresses IL-12 expression in DC

**Figure 1:**
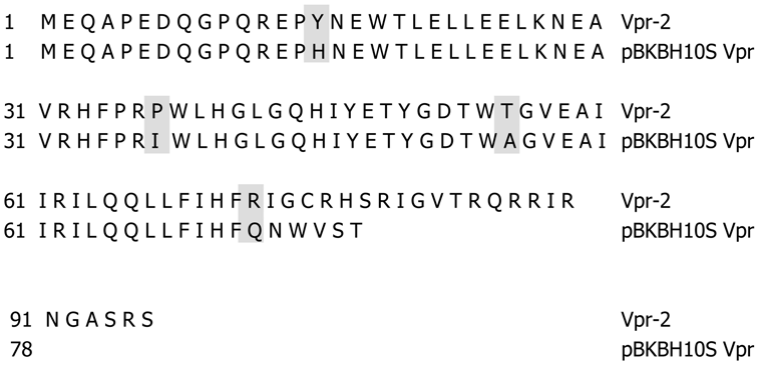
Alignment of amino acid sequence of Vpr amplified from isolate Vpr-2 and non infections plasmid pBKBH10S. Alignment and alignment report were generated using Lasargene software (DNAstar). Amino acids 15, 37, and 55, outside of the C-terminal region which differ between the two Vpr proteins are highlighted by grey boxes. The last grey box (amino acid 74) indicates the divergence of the two sequences at the C-termini.

The first observation of Vpr-mediated IL-12 suppression was made in a side-by-side comparison of DC electroporated with Vpr amplified from two different sources; Vpr amplified from the non-infectious clone, pBKBH10S [20] did not have any negative impact whereas Vpr amplified from a patient's plasma (Vpr-2) led to a several-fold suppression of IL-12 secretion. Alignment of the Vpr sequences used in this initial experiment revealed that the amino acid sequence of Vpr allowing secretion of IL-12 in the culture was truncated at its C-terminus (**Figure 1**).

**Figure 2:**
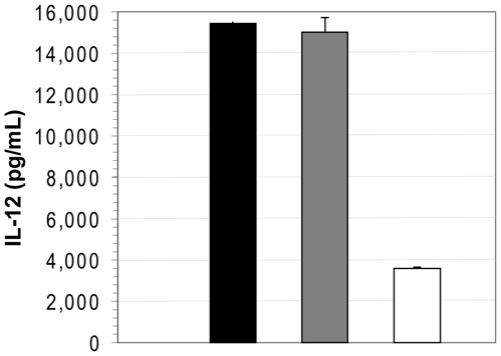
Full length but not truncated Vpr inhibits IL-12 secretion by DC. Levels of IL-12 detected in supernatants of DC cultures 24 hrs post-electroporation. Black bar GFP, grey bar: 1 µg of RNA of Vpr truncated version pattern bar: 0.25 µg of full length Vpr (Vpr 2) were electroporated per million of DC. Condition when GFP RNA was electroporated alone is taken as maximum potential level of IL-12 cytokine secretion in each experiment.

In order to confirm this initial observation and rule out the possible contribution of other previously reported suspect amino acids in positions 15, 37, and 55, the experiment was repeated using full-length and truncated Vpr generated from a single template (Vpr-2), as described in materials and methods. The levels of secreted IL-12 were measured in supernatants of DC electroporated with either full-length or truncated Vpr RNAs (**Figure 2**). In this experiment, IL-12 secretion in the supernatants of DC electroporated with RNA encoding full-length VPR (0.25 µg per 10^6^ DC) is suppressed by 75% compared to those electroporated with control RNA encoding GFP. Also, electroporation of DC with even higher amounts of truncated Vpr RNA (1 µg per million of DC) did not result in substantial suppression of IL-12 secretion. Since electroporation with a lower amount (0.25 µg per 10^6^ DC) of full-length Vpr resulted in substantial inhibition of IL-12 secretion, a higher amount of RNA (*e.g.*, 1 µg per 10^6^ DC) was not tested in this experiment.

The absolute level of IL-12 secreted into the supernatant is highly variable among independent healthy donor cells [21], [22]. To control for this variability, the data in each experiment are interpreted as a relative level to the control condition where cells were electroported with the inert RNA encoding GFP. These data confirm that full-length Vpr suppresses IL-12 expression and the domain responsible for the inhibition is located within amino acids 79–96.

### Vpr expressed from transfected RNA does not lead to maturation defects in DC

Previous reports suggested that Vpr blocks the maturation status of the DC as indicated by the change in expression of the surface phenotype markers CD80, CD83 and CD86 [11], [12]. Expression of these surface markers as well as viability were examined in DC electroporated with low (0.25 µg) or high (1 µg) masses of Vpr RNA at per 10^6^ DC (**Table 1**).

**Table 1:** Cell surface phenotype profiles of mDC transfected with Vpr RNA.

RNA mass (µg/10^6^ DC)	% CD80*	% CD83*	% CD86*	Viability**
1 µg eGFP control	87	84	100	100
1 µg VPR, trunc	96	94	100	104
0.25 µg VPR, trunc	96	94	100	104
0.25 µg VPR, full	96	91	100	96

* Cell surface phenotype of the DC is expressed in percent positive cells staining with the indicated antibody.

** Viability at 24 hrs post electroporation time point is expressed in percent relative to control DC transfected with eGFP RNA alone.

**Figure 3:**
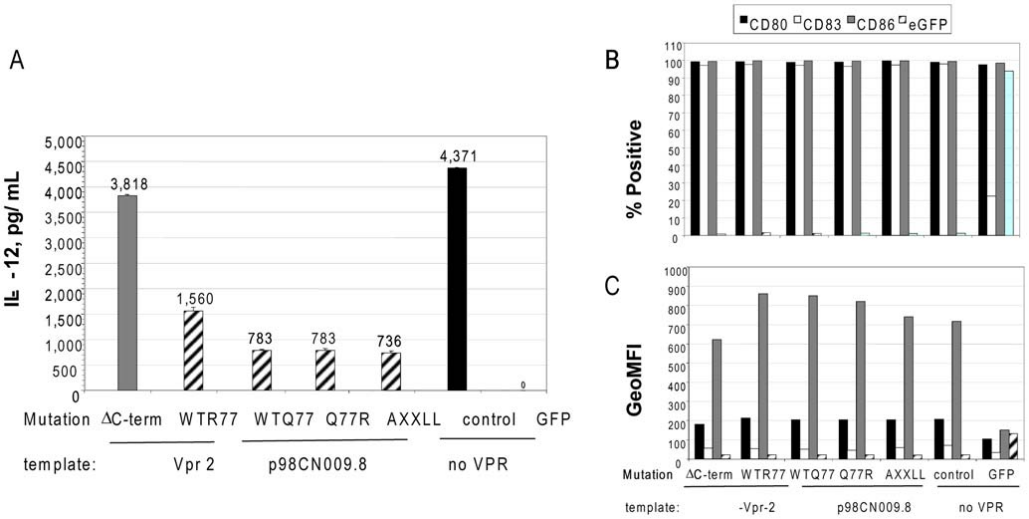
Effect of mutants on IL-12 secretion and immunophenotype. Analysis of IL-12 secretion profile and phenotype of the DC transfected with Vpr mutants in position 77 and LXXLL C-terminal motif. Full-length Vpr from either Vpr 2 or p98CN009.8 plasmid or its mutants, no Vpr with CD40L RNA (control) or GFP alone were electroporated into DC at amount of one microgram of RNA per million of DC. Panel A: levels of IL-12 cytokine detected in DC supernatants 24 hrs post- electroporation. Panel B: percent positive cells stained with specific anti CD80, anti CD83 and anti CD86 antibody. Panel C: mean fluorescent intensity of signal detected with specific antibody.

The mean fluorescent intensity for staining with the antibodies correlates with the level of protein expressed per cell and did not vary between the different conditions in this experiment (data not shown, detailed analysis presented in **Figure 3**).

These experiments demonstrate that phenotypic maturation markers expressed on DC are not affected when Vpr is expressed from translation-competent RNA. This observation is inconsistent with previous reports [11], [12]. The difference may be due to the different platforms utilized for Vpr expression. This study utilized translation-competent Vpr RNA under conditions that optimize cytoplasmic delivery whereas Majumder and colleagues employed HIV-1 *Vpr+* or HIV-1 *Vpr−* viruses for the infection of the DC [12] and Muthumani and colleagues used cultures treated with purified recombinant Vpr protein [11].

### Amino acid R77 and the LXXLL domain are not responsible for suppression of IL-12 cytokine secretion

The data described above demonstrates that the Vpr C-terminal domain is responsible for the IL-12 suppressive effect and provides an initial analysis of which amino acids may be responsible for the IL-12 blockade with emphasis on those already described in literature. Argenine in position 77 of Vpr is reported to bind to adenine nucleotide translocator (ANT) expressed in the mitochondrial membrane causing its depolarization which leads to the induction of a caspase-dependent pathway and eventual apoptosis. At least two reports link the R77Q substitution in Vpr with long-term non-progression (LTNP) of HIV-infected patients [13], [18]. The HIV clone, p98CN009.8, contains Vpr with a Glutamine (Q) in position 77 and we used it to prepare Vpr RNA encoding Q77. In addition, we created the reverse substitution, Q77R in the p98CN009.8 background. If the R77 residue is responsible for the IL-12 suppression it is expected that Q77 would relieve the IL-12 suppression and the reverse mutation Q77R will have the opposite effect.

We also evaluated the contribution of the LXXLL domain found within the C-terminus of Vpr. The GR-like LXXLL motif is implicated in suppression of IL-12 production in monocytes[19]. This study reported that through the involvement of the LXXLL coactivator signature motif, Vpr acts as a potent coactivator of the glucocorticoid receptor pathway and IL-12 is one of the targets regulated by that pathway. The core LXXLL motif in ligand-bound nuclear hormone receptors is necessary and sufficient to bind the cellular transcriptional co-activator proteins, a function known to be disrupted by the AXXLL mutation [23]. An AXXLL containing Vpr mutant was created in the background of the p98CN009.8 *Vpr*.

To test the impact of these mutations on the ability to regulate IL-12 secretion by DC, all of the RNAs amplified from Vpr mutants were transfected into DC. IL-12 expression profiles in the supernatants of the transfected DC is presented on **Figure 3**, panel A. Analysis of cell surface marker expression is presented in **Figure 3**, panels B and C. Cytokine levels in DC culture supernatants collected 24 hrs post-electroporation indicate that RNA encoding the truncated Vpr amplified from template Vpr-2 do not inhibit IL-12 secretion and is similar to the control condition where cells were electroporated with only control GFP RNA. As previously observed, truncated Vpr amplified from Vpr-2 relieved the IL-12 suppression. The full-length Vpr RNA amplified from p98CN009.8 also suppressed the IL-12 secretion. This indicates that Arginine in position 77 is not involved in the regulation of the IL-12 cytokine pathway. Since amino acid 77 is not involved, it was not surprising that the reverse mutant Q77R did not differ from the wild-type p98CN009.8 and suppressed IL-12 secretion as well.

Our observation that disruption of the core LXXLL motif did not result in suppression of IL-12 secretion is in contrast to reported observations using purified Vpr protein in monocyte cultures. The observed differences of IL-12 levels may be due to the differences in the two experimental systems. This experiment also confirmed our original observation that the MFI of the maturation markers, CD80, CD83, and CD86 are not significantly different from the GFP RNA control, (**Figure 3**, panel B), nor did the percentage of cells expressing those markers differ between each CD40L DC-matured population (**Figure 3**, panel C). Expression of AXXLL and R77Q Vpr RNAs was confirmed in vitro (Data not shown). In addition continuous suppression of IL-12 in the DC electroporated with the AXXLL and R77Q Vpr RNA indirectly suggests lack of those mutations on the expression of Vpr in DC. The lower levels of maturation markers expressed by the GFP RNA-electroporated control cells was due to the lack of co-transfection with CD40L RNA, which is required to achieve full DC maturation in this system.

### The C-terminal domain structure is important for Vpr-mediated suppression of IL-12 secretion by DC

**Figure 4:**
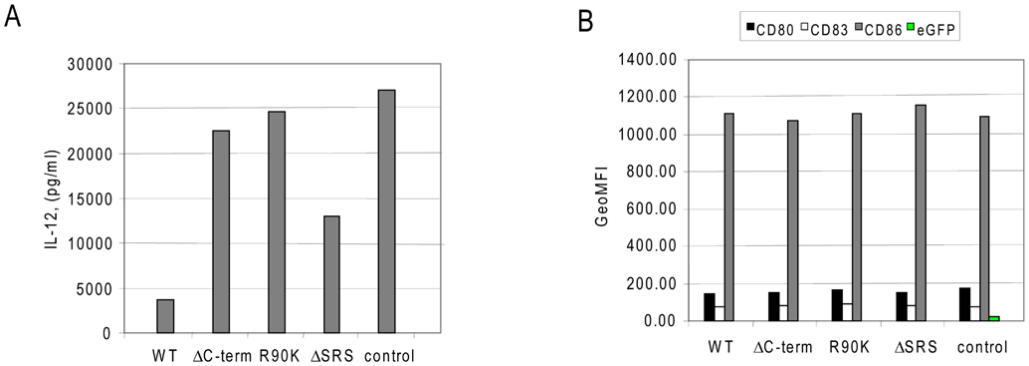
Vpr R90K mutation restores IL-12 secretion from DC. Analysis of IL-12 secretion profile and phenotype of the DC transfected with Vpr mutants in C-terminal region. Full-length Vpr from p98CN009.8 plasmid or its mutants, or no Vpr with CD40L RNA (control) were electroporated into DC at amount of one µg of RNA per million of DC. Panel A: levels of IL-12 cytokine detected in DC supernatants 24 hrs post- electroporation. Panel B: mean fluorescent intensity of signal detected with specific antibody recognizing CD80, CD83, and CD86 surface markers. Viability of cells 24 hrs post electroporation with WT, Δ−C term, R90K ΔSRS or GFP RNAs were 93%, 93%, 94% 94% and 94% respectively.

Given than none of the mutations tested above demonstrated any impact on IL-12 secretion, we next created a new series of mutations within the C-terminal region: R90K as well as deletion of the last three amino acid residues, eliminating both S94 and S96 (ΔSRS). These residues are reported to be important for transactivation of Vpr and induction of G2 cell cycle arrest (reviewed in [24]). The R90K and ΔSRS mutants were created in the background of the p98CN009.8 clone. The RNAs transcribed from these cDNA fragments were electroporated into DC followed by an IL-12 Elisa assay on 24 hr supernatants. As a control the RNAs were electroporated in the absence of any Vpr RNA (**Figure 4**). In this experiment the use of the C-terminal truncated form of Vpr versus full-length (WT) Vpr (1 µg of RNA per 10^6^ DC) demonstrated relief of IL-12 suppression. More interestingly, cultures of DC electroporated with R90K Vpr had levels of IL-12 comparable to those observed using the C-terminal Vpr truncation or in the complete absence of Vpr RNA (GFP control). The same result was obtained with p98CN009.8 Vpr RNAs and its mutants transfected into DC at 5 µg RNA per 10^6^ DC (data not shown). The data also repeated with all Vpr mutants created in the Vpr-2 genetic background (data not shown). The viability of the DC cultures post electroporation was 93–94% for all conditions in this experiment; therefore the different IL-12 levels measured in the supernatants were not due to different numbers of viable cells in the cultures. The percent of viable dendritic cells vary from experiment to experiment because of different donor material used to generate the DCs. Therefore the viability data should be derived from intra assay conditions. These data clearly show that a single conservative amino acid substitution, R90K, in full length Vpr prevents the IL- 12 blockade in DC.

### C-terminal deletion or R90K mutation leads to Vpr protein degradation via ubiquitin-dependent pathway

**Figure 5:**
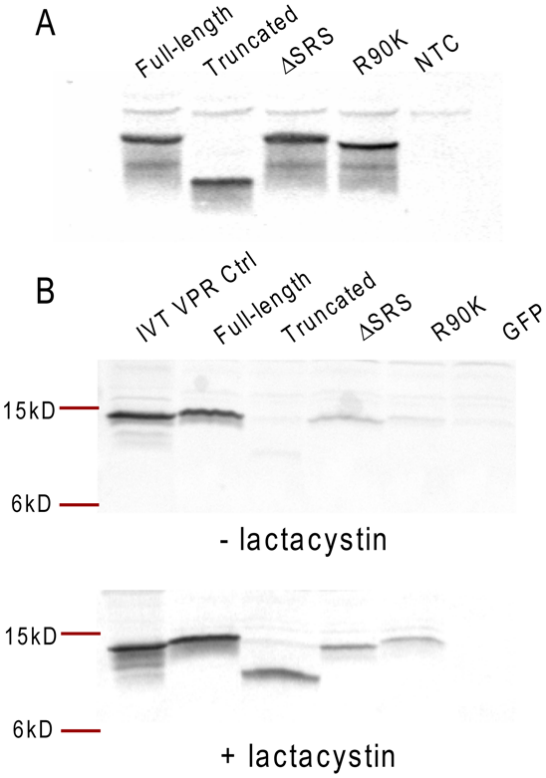
Vpr mutants have enhanced ubiquitin-mediated degradation leading to low steady-state levels in DC. Analysis of Vpr expression from RNA template *in vitro* and in DC. Panel A:Western blot analysis of *in vitro*-translated full-length Vpr RNA or its mutations as indicated. NTC: control reaction performed in the absence of an RNA template. Panel B: Western blot analysis on lysates of DC electroporated with full length Vpr RNA or its mutations. After electroporation DC were incubated for four hours in the absence or presence of lactacystin at 10 µM final concentration as indicated. IVT VPR Ctrl: *in vitro*-translated full-length Vpr protein used as a positive control in Western blot assay. RNAs used for electroporation of DC are indicated on the top.

In order to confirm that proteins were expressed from wild-type, truncated and R90K mutant RNAs we conducted studies using Western blot analysis. First, translation of Vpr products was confirmed using an *in vitro* translation system. The Western blot analysis results indicate that all RNAs produced polypeptide products *in vitro* migrating at an expected molecular weight and recognized by a specific anti-Vpr polyclonal antiserum (**Figure 5**, panel A). These data indicated that the presence of the C-terminal truncation or R90K mutation do not impact on the ability of the ribosomal machinery to translate Vpr RNAs.

The next experiment analyzed the Vpr protein expression levels in DC that were electroporated with 5 µg per 10^6^ DC of each Vpr RNA. A higher mass of RNA was chosen because of the low sensitivity of the Western blot analysis and the lack of a strong signal obtained with the lower mass of 1 µg of RNA per 10^6^ DC (data not shown). The IL-12 expression data obtained with either 1 or 5 µg of RNA were comparable. A specific signal was obtained in DC lysates transfected with the full length Vpr (**Figure 5**, panel B) however, the expression levels of the truncated Vpr or R90K mutant are decreased dramatically by comparison. Since all of the proteins were efficiently translated *in vitro* (**Figure 5**, panel A), the differences in the expression levels in DC cannot be explained by the differential translational competence of the RNAs. To further explore this, we repeated the analysis and allowed the DC to recover post-electroporation in the presence of a ubiquitin degradation pathway inhibitor, lactacystin (**Figure 5**, panel C). In the presence of lactacystin, there was an accumulation of the Vpr-specific signals using lysates of DC electroporated with truncated Vpr or the R90K mutant. Although all Vpr RNAs are translated in the DC, both the truncated form and the R90K mutant are rapidly degraded and the lack of their expression persistence may explain their non-interference with IL-12 secretion. In the presence of the inhibitor of ubiquitin-mediated degradation, the levels of the truncated form of Vpr and the R90K mutant are elevated.

### The R90 residue is highly conserved in Vpr and R90K is the only substitution tested which relieves the IL-12 suppression

Next we examined the frequency of amino acid substitutions at position 90 of the Vpr protein in the Los Alamos HIV sequence database. Full-length Vpr protein sequences (n = 1,226) were aligned and the identity and frequency of the amino acid in position 90 was examined (**Table 2**). At the time of this analysis, the Los Alamos sequence database did not list any Vpr protein with K (lysine) in a position 90. 98.78% of all analyzed sequences contained R (arginine) in position 90 indicating that this amino acid is highly conserved. The frequency of amino acid substitutions in position 90 is less than 0.5%.

**Table 2:** Analysis of frequency and type of amino acid substitutions in position 90 of 1226 full length Vpr sequences found in Los Alamos National Database.

Amino acid in Vpr position 90	Total number reported	%. relative to total number of analyzed sequences
R	1211	98.78
G	6	0.49
S	3	0.24
A	2	0.16
T	2	0.16
Q	1	0.08
I	1	0.08

**Figure 6:**
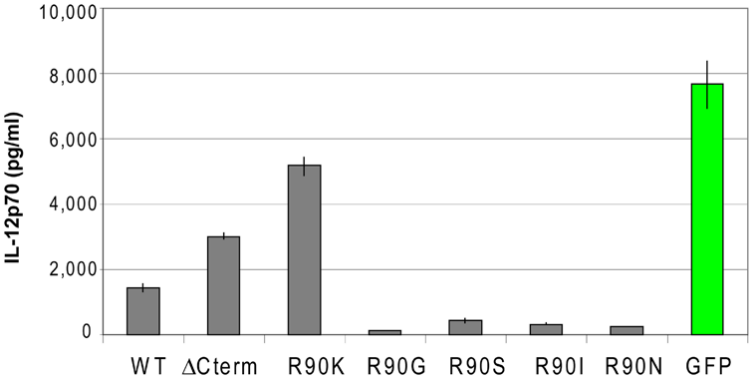
Analysis of amino acid substitutions reported in Los Alamos database on Vpr ability to modulate IL-12. Vpr clones carrying the most frequently reported amino acid substitutions in position 90 of 1,226 full-length Vpr sequences in the Los Alamos National Database were cloned and tested for their ability to modulate IL-12 secretion by the DC. Vpr wild-type (WT), VPR lacking C-terminus (ΔCterm), Vpr R90K, Vpr R90G, Vpr R90S RNAs or GFP RNA alone were electroporated into DC and levels of IL-12 detected in DC supernatants 24 hrs post-electroporation. Viability of cells electroporated with WT and mutant Vpr RNA was 71–85% whereas the viability of a GFP RNA-electroporated DC was 76%.

The impact of all amino acid substitutions reported in the Los Alamos HIV sequence database at Vpr position 90 were next examined in the IL-12 secretion assay. The RNA encoding the two most frequently reported substitutions, G and S, in the background of p98CN009.8 were created and transfected into DC followed by measurement of IL-12 secretion by Elisa. R90 and R90K were also used in the same experiment as controls (**Figure 6**). Of all amino acid substitutions tested in position 90, only the R90K relieves the suppression of IL-12. Interestingly, Vpr RNA encoding for amino acids substitutions G and S in position 90 suppress IL-12 secretion to levels comparable or even lower than the wild-type R90 Vpr.

## Discussion

HIV Vpr has been shown to negatively regulate the function of antigen presenting cells such as macrophages and dendritic cells [11], [12]. Our study, using a very different expression system, translation competent RNA, independently confirms the observation that Vpr blocks production of the Th1 cytokine, IL-12, which is required for CTL activation [8], [9]. We also demonstrate that upregulation of phenotypic cell surface markers of DC maturation (i.e., CD86, CD80, CD83) in the absence of IL-12 secretion indicates that upregulation of those molecules alone is not sufficient for the induction of IL-12 secretion. Levels of IL-12 are decreased in HIV-positive individuals versus healthy volunteers [7] and Th-2 cytokines predominate in the plasma of late-stage AIDS patients [10]. The reduced cellular immunity may be explained by the blocking of IL-12 secretion by antigen presenting cells such as DC expressing Vpr. This idea is supported by a study in rhesus macaques where inclusion of the Vpr accessory gene in an experimental plasmid-based vaccine containing Nef antigen markedly reduced Th1 type responses compared to the vaccine containing only the Nef antigen, which led to decreased CD4 T cell counts and increased viral loads [25].

Negative effects attributed to Vpr manifested by downregulation of costimulatory molecules and suppression of IL-12 secretion have been reported. In contrast to previous reports, our study did not confirm the observation of CD83, CD86, and CD80 downregulation. This may be the due to the difference in delivery system of Vpr to the DC. The study by Muthumani *et. al* [11] used Vpr recombinant protein which was co-incubated with the DC and Majumder *et. al.* used DC transfected with HIV Vpr+ proviral constructs [12] whereas in our study Vpr expression is achieved using translation-competent RNA electroporated into the DC. The Vpr RNA is translated in the cytoplasm of the DC and is a highly homogeneous expression system which bypasses the need for Vpr RNA transcription and may possibly deliver the functional Vpr to different subcellular compartments compared to other expression methods or the provision of exogenous Vpr protein. As a consequence, variable effects may be observed.

Detailed analysis of the C-terminal region did not support any previously reported mutations in the C-terminal region as modulators of IL-12 expression levels. Mirani *et al*, desribed downregulation of IL-12 production via a GR-like function in cultured monocytes by adding purified recombinant Vpr protein and modulating the effect on IL-12 with the GR antagonist, RU486 [19]. The direct Vpr GR-like effect on IL-12 secretion in our system was elucidated by mutating the LXXLL motif to AXXLL which is reported to inactivate the nuclear receptor function. However, side-by-side comparison of the DC electroporated with two Vpr RNAs encoding either a functional LXXLL motif or containing the inactivating mutation, AXXLL, did not demonstrate any difference in the ability to express the IL-12. Additionally, the R77Q mutation of Vpr is thought to be associated with long-term control of HIV infection [13], [14]. If preservation of IL-12 expression is associated with the LTNP phenotype, R77Q was a good candidate for testing in the IL-12 secretion assay. However, our data show that in a direct comparison of RNA encoding wild-type R77 and the Q77 mutant, in otherwise identical Vpr amino acid backgrounds, no difference in IL-12 secretion is observed.

Interestingly, a high rate of Vpr mutations and other defects in the C-terminal domain are described in LTNP cohorts [26]–[28]. The deletion of the last three amino acids did not change the ability of Vpr to modulate IL-12 secretion, however, the R90K substitution revealed complete reversal of IL-12 suppression, restoring the level to that observed with the truncation of the entire C-terminal Vpr domain. Interestingly, sequence analysis of Vpr within the Los Alamos Database revealed no such mutants. The analysis revealed that other amino acid substitutions such as R90G and R90S are present, but at a cumulative frequency not exceeding 1.3%. Reports of the R90K substitution were found by a manual search of the literature [26], [29] and in both cases this mutation was isolated from a patient exhibiting an LTNP phenotype. It is therefore possible to envision that strains of HIV encoding R90K have lower virulence. Virus encoding Vpr R90K may be unable to block IL-12 secretion. IL-12 cytokine is a component of LTNP phenotype in HIV infected individuals, and is a common feature with healthy individuals. Our analysis of amino acids reported at position 90 indicated that R (arginine) is highly conserved. None of the amino acids reported in this position tested in our assay relieve IL-12 suppression and the R90K substitution is the only substitution which restored the Il-12 secretion profile. These data taken together suggest that HIV naturally selects for a sequence encoding for amino acids which suppress IL-12 secretion by DC and against the mutation(s) which relieves this suppression.

NMR structural analysis of the C-terminal region of Vpr suggests it has a dimeric structure [30], [31] which is confirmed experimentally [30]. Studies of the C-terminus predicted the dimeric structure of the full length Vpr protein in solution and the dimerization was postulated to be biologically relevant. Our data demonstrate that the intact C-terminus is important for the suppression of IL-12. It would be of interest to explore whether the R90K amino acid substitution causes disruption of the Vpr dimeric structure. The amino acids previously described by Schuler *et al* resulting in the disruption of the dimers do not involve amino acid position 90 [30]. Our study uncovers the mechanism of how the R90K substitution allows for IL-12 secretion in the DCs.The R90K substitution perhaps is recognized as a defect and directs Vpr for rapid degradation. This would suggest that the R90K substitution is not directly involved in activation of the IL-12 secretion pathway; rather the R90K substitution alleviates the suppressive effect of wild-type and other amino acids found in this position through the reduction of intracellular Vpr protein levels. In summary, our study documents a single amino acid substitution which is not found in most of the reported HIV genomes. We predict that selective pressure exists to preserve an amino acid at position 90 which supports IL-12 suppression. The R90K substitution relieves IL-12 suppression and is selected against, since other amino acids more distantly related to R than K are observed in infected subjects. This finding provides further support for Vpr as a virulence factor during HIV infection. The DC matured in the presence of Vpr are defective in the production of IL-12 and eventually lose their ability to orchestrate productive T cell responses thus mediating, at least in part, the switch from Th1 to Th2 cytokine production which is a hallmark of progression to AIDS.

The physiologic contribution of Vpr to natural viral infection and persistence is perplexing. The negative influence of IL-12 blockade on adaptive antiviral immunity is in apparent conflict with studies which highlight Vpr as a dominant immune target. Cytotoxic CTL responses against Vpr in HIV infection are well documented [32] and Vpr is described as an antigen to which CTL responses are preferentially directed [33]. We recently reported a novel multiplex RT-PCR-based approach for the generation of autologous HIV antigens, including Vpr, for the immunotherapy of HIV-infected individuals [34]. Our experiments demonstrated Vpr-specific stimulation of CD8 T cell proliferation by DC expressing Vpr [34]. However, the PCR-amplified portion of Vpr used in that study lacked the C-terminal 20 amino acids, thus allowing Vpr expression without IL-12 suppression. The data presented in this manuscript provides a molecular basis for the generation of anti-Vpr CD8^+^CD28^+^CD45RA^−^ effector memory T cells, which is known to require IL-12 secretion [35]. Importantly, for immunotherapeutic design, the truncation of Vpr protein to amino acids 1–72 preserves all currently described HLA epitopes documented in the HIV immunology database (http://www.hiv.lanl.gov/content/immunology/maps/ctl/Vpr.html) located between amino acids 7 and 70 and does not compromise the ability of the DC expressing the truncated Vpr to secrete IL-12. The ability to utilize Vpr as a component of experimental immunotherapeutics may be particularly important. since a systematic bioinformatic analysis of the Clade C HIV proteome revealed that HIV-1 mutations in Vpr which confer escape from CTL recognition can result in the lowest viral fitness compared to those directed against any other HIV protein [2]. These observations should figure prominently in the choice of antigens for use in anti-HIV immunotherapeutic design and consideration should be given to preserving or restoring IL-12 secretion.

## Materials and Methods

### Cellular materials used to generate the DCs

All materials used in the studies described in this paper collected from healthy volunteers at Lifeblood Biological Services, LLC. The company operates under FDA establishment license to collect normal subjects for products for research use only. The collection is preceded by signing of a written consent form.

### Generation of DC and cellular analyses

Generation, maturation, and electroporation DC from healthy volunteers were carried out according to the CD40L DC maturation protocol described by Calderhead *et. al* [36]. The electroporated mass of RNA is indicated in each experiment as µg per million DC. The phenotype of the DC were evaluated by staining with phycoerythrin (PE)-conjugated mouse anti-human antibodies: CD80 (L307.4), CD83 (HB15e), and CD86 (FUN-1). Isotype matched mouse IgG1 (MOPC-21) was used as a negative control. All antibodies were sourced from BD Biosciences, San Jose, CA. Cell staining and Enzyme-linked Immunosorbent Assay (ELISA) for IL-12 levels in DC supernatants was carried out as previously described [36] Cells electroporated with each RNA were incubated in duplicates and measurements were performed in undiluted and diluted supernatants. Values were obtained using all conditions falling within the IL-12 detection range corrected for the dilution factor.

Cell viability was determined using Caltag Counting Beads (Invitrogen) and propidium iodide (to identify dead cells) both pre- and post-transfection. Cells were diluted to 1×10^6^ cells/ml following transfection and incubated overnight. Cells were harvested 18–22 hours post electroporation and supernatants used for IL-12 determination while the cells were stained for the maturation markers CD80, CD83 and CD86.

### Analysis of Vpr translation products by Western Blot analysis

*In vitro*-translated products of Vpr RNA were generated using a Wheat Germ Lysate translation system (Promega). Analysis of Vpr translation was by Western blot with DC lysates prepared from cells electroporated with Vpr or control RNA after 4 hours post-electroporation with or without lactacystin at 10 mM final concentration. Total protein was extracted from DC using M-PER mammalian protein extraction reagent (Pierce) according to manufacture's recommendations. Protein concentration was determined by the Bradford assay method (Bio-Rad). 40 µg of total protein extract from DC or a 1 µL aliquot of *in vitro* translation reaction product were separated by SDS PAGE and transferred to PVDF membrane. The membrane was incubated with a 1∶500 dilution of Rabbit polyclonal antisera HIV-1_NL4–3_ Vpr Antiserum (1–40) from the NIH AIDS Research & Reference Reagent program followed by incubation with Peroxidase-labeled anti-rabbit secondary antibodies (Amersham). The signal was developed using ECL Plus reagents (Amersham) and scanned using a Storm imager.

### Amplification of Vpr RNA from infectious plasma

RNA extraction from plasma and amplification of sequences containing the coding region of Vpr were performed as described previously [34]. The primary PCR product was taken into a secondary PCR reaction containing primers modified by insertion of a T7 promoter sequence and a 64T nucleotide sequence. Generation of full-length Vpr or its truncation was performed using alternative reverse primer groups. The primers anneal to upstream (64T T trunc) or downstream (64T full) region of naturally occurring the Vpr Stop codon, thus leading to amplification of truncated or full length Vpr, respectively.

Sequences of Vpr primers used in the secondary PCR reaction are listed in **Table 3**.

Amplification of Vpr from a non-infectious clone, pBKBH10S, was performed using single forward and reverse primers complimentary to the template determined by sequence analysis. [20].

**Table 3:** Sequences of Vpr primers used in the secondary PCR reaction to generate full length or truncated at C terminus Vpr.

Name	5′–3′ sequence
T7 F 1	TAATACGACTCACTATAGGGAGACCACCATGGAACAAGCCCCAG
T7 F 1.1	TAATACGACTCACTATAGGGAGACCACCATGGAACAAGCCCCGG
64T full 1	(T)_64_GCAGTTGTAGGCTGACTTCC
64T full 1.1	(T)_64_GCAGTTGTAGGCTGACTCCC
64T full 1.2	(T)_64_GCAGTTGTAGGCTGGCTTCC
64T trunc 1	(T)_64_AGCGAACAAACAGTAGTTGTTGCAG
64T trunc 1.1	(T)_64_AGCGAACAAACAGTAGTTGTTGCAA
64T trunc 1.2	(T)_64_AGCGATCAAACAGCAGTTGTTGCAG
64T trunc 1.3	(T)_64_AGCGAACAAACAGTAGTTGTTGAAG
64T trunc 1.4	(T)_64_AGCGATCAAACAGTAGTTGTTGCAG

### Generation of C-terminal mutant clones of Vpr

Mutations in position 77 were generated using the non-infections plasmid, p98CN009.8, containing the near full-length HIV genome [37]. This clone contains a naturally occurring Q77 residue. The Q77R amino acid substitution was constructed using PCR amplification with primers encoding the mutation listed in **Table 4**. The PCR fragment amplified from the p98CN009.8 template was gel purified and subcloned into a pUC19 vector.

**Table 4:** Sequence of primers used to construct Vpr encoding for the Q77R amino acid substitution.

Name	5′–3′ sequence
5′ VPR BamHI:	CTGAGGATCCGGGAGACCACCATGGAACAATCCCCAGAA
3′ VPR Q77R Pst I	TACTCTGCAGCCTTCAGTCGGATGTTGACGTTAGGATCTACTGGCTCCATTTCTTGTTCTTCTCTGTCTCAAAATGCCTATTCTGCTATGCCGACACC

The AXXLL mutation was introduced by a fusion PCR approach. First, the template plasmid, Q77R p98CN009.8, was amplified using 2 PCR reactions with the four primers listed in **Table 5**. The mixture of the two PCR fragments served as a template for the second PCR reaction using primers 5′ VPR BamHI and 3′ VPR Pst I only (**Table 5**). The fragment was subcloned into a pUC19 vector. The presence of all mutations was verified by sequence analysis.

**Table 5:** List of primers used in a fusion PCR approach to create AXXLL Vpr construct.

Name	5′–3′ sequence
5′ VPR BamHI	CTGAGGATCCGGGAGACCACCATGGAACAATCCCCAGAA
VPR L64A Reverse	ACAGCAGTTGTTGGGCAGTTCT
3′ VPR PstI	TACTCTGCAGCCTTCAGTCGGATGTTGACGTTAGGATCTACTGGCTCC
VPR L64AForward	AGAACTGCCCAACAACTGCTGT

### In vitro transcription

PCR fragments containing T7 Promoter sequences or linearized pUC19 plasmid containing various Vpr mutants were *in vitro*-transcribed using a mMessage mMachine T7 Ultra kit (Ambion) according to the manufacturer's recommendation. The RNA was purified using RNeasy columns (QIAGEN) and eluted in water. The RNA was stored frozen in liquid nitrogen in single use aliquots.

### Analysis of frequency of amino acid substitution in a position 90 of Vpr proteins

Amino acids of C-terminus of Vpr protein were aligned using HIValign tool at Los Alamos HIV sequence database using 7 amino acids as a query (http://www.hiv.lanl.gov/content/sequence/HMM/HmmAlign.html) total number of returned aligned sequences was 1,226. The identity and frequency of amino acid substitutions in position 90 were calculated using Microsoft Excel.
